# A Fibroid or Cancer? A Rare Case of Mixed Choriocarcinoma and Epithelioid Trophoblastic Tumour

**DOI:** 10.1155/2013/492754

**Published:** 2013-05-02

**Authors:** Wan Yu Luk, Michael Friedlander

**Affiliations:** ^1^Royal Hospital for Women, Barker Street, Randwick, NSW 2031, Australia; ^2^Gyneacological Cancer Centre, Royal Hospital for Women, Randwick, NSW 2031, Australia

## Abstract

*Background*. Gestational trophoblastic disease (GTD) is a rare complication of pregnancy which is characterised by abnormal growth of the trophoblasts at the placental site. It is categorised into benign and malignant forms, which include hydatidiform moles (HMs) and gestational trophoblastic neoplasia (GTN), respectively. A mixed choriocarcinoma (CC) and epithelioid trophoblastic tumour (ETT) is an extremely rare subgroup of GTN, which is a highly curable but aggressive form of malignancy. *Case*. We report a case of mixed CC and ETT in a 41-year-old patient who presented with a 2-year history of menorrhagia and fibroid uterus in the absence of previous history of molar pregnancy. She had a 12-year interval between the antecedent pregnancy and presentation. She was treated with intensive regimen of adjuvant chemotherapy, etoposide, methotrexate, and actinomycin-D with etoposide and cisplatin (EMA-EP). She has remained disease free for more than 5 years. *Conclusion*. This case highlights the importance of considering GTN as one of the differential diagnoses value of **β**-HCG in patients presented with menorrhagia and growing fibroids.

## 1. Introduction 

Gestational trophoblastic disease (GTD) is a rare complication of pregnancy. GTD is characterised by abnormal growth of trophoblasts at the placental site. It is categorised into hydatidiform moles (HMs) and gestational trophoblastic neoplasia (GTN). The former subgroup is usually benign and treated by dilatation and curettage to evacuate the abnormal placental growth. However, persistent HM can become malignant and transform into GTN. The latter subgroup represents the malignant forms of the disease which includes invasive mole, choriocarcinoma, placental-site trophoblastic tumour (PSTT), and epithelioid trophoblastic tumour (ETT). A mixed choriocarcinoma (CC) and epithelioid trophoblastic tumour (ETT) is an extremely rare and aggressive form of GTN, but highly curable with good prognosis if detected early [1–3].

## 2. Case Report

A 41-year-old presented with a 2-year history of menorrhagia. She has had three normal pregnancies and deliveries. Her husband had a vasectomy 12 years ago. There was no other known pregnancy. In addition, she reported a few-month history of nausea and engorged breasts at presentation. All previous Pap smears were normal. She was otherwise healthy with no significant medical and family history. 

Physical examination of the abdomen showed a palpable uterus but no other masses. The pelvic ultrasound showed a moderately bulky fibroid uterus with an endometrial thickness of 5 mm. Despite the insertion of Mirena, she reported further increase in menstrual bleeding. Repeated pelvic ultrasound showed an increase in size of the fibroids and endometrium thickness up to 8.3 cm. 

A total abdominal hysterectomy (TAH) was performed 7 months later. The uterus was noted 16 weeks gestation in size but otherwise normal in appearance. Histopathology confirmed a mixed CC and ETT. The serum *β*-HCG level was markedly raised to 2,223 one week post-TAH. Other serum tumour markers including CEA, Ca 125, and Ca 19.9 were all within the normal range. The staging imaging showed no evidence of metastatic disease. She was commenced on adjuvant chemotherapy with an intense regimen alternating etoposide, methotrexate and actinomycin-D with etoposide and cisplatin (EMA-EP) and had 5 cycles of treatment. She was monitored closely with regular *β*-HCG surveillance and serial CT scans. She has been disease free for more than 5 years.

## 3. Discussion

The incidence of GTD varies worldwide due to the variation of definition and method of diagnosis [4]. The reported incidence of GTD in Australia and New Zealand is 74 per 100,000 pregnancies [1, 4, 5]. The age-standardised incidence of CC in the same region is less than 0.13 in 100,000 pregnancies. A mixed CC and ETT is an extremely rare form of GTD. The two established risk factors for GTD are extremes of reproductive age (<20 and >40 years) [2] and previous molar pregnancy [1–5]. Patients commonly present with uterine bleeding and a rapidly enlarging uterus with a history of previous GTD. Despite the association in pregnancy, the interval between the antecedent pregnancy and presentations can be many years as illustrated by this case. GTD can also affect peri- and post-menopausal women [3]. Therefore, GTD should be considered as part of the differential diagnosis in women with vaginal bleeding and a uterine mass. *β*-HCG and pelvic ultrasound are disease-specific and remain the gold standards for diagnostic evaluation of GTD. Hysterectomy and adjuvant chemotherapy are the current recommended management for epithelioid trophoblastic tumour.

This case report highlights the diagnostic value of *β*-HCG in the context of menorrhagia and growing fibroids with no previous molar pregnancy. 
